# *Caesalpinia decapetala* Extracts as Inhibitors of Lipid Oxidation in Beef Patties

**DOI:** 10.3390/molecules200813913

**Published:** 2015-07-31

**Authors:** Maria G. Gallego, Michael H. Gordon, Francisco J. Segovia, María P. Almajano

**Affiliations:** 1 Chemical Engineering Department, Universitat Politècnica de Catalunya, Av. Diagonal 647, 08028 Barcelona, Spain; E-Mails: maria.gabriela.gallego@upc.edu (M.G.G.); segoviafj@gmail.com (F.J.S.); 2 Department of Food and Nutritional Sciences, University of Reading, Whiteknights, P.O. Box 226, Reading RG6 6AP, UK; E-Mail: m.h.gordon@reading.ac.uk

**Keywords:** *C. decapetala*, antioxidant, polyphenol, free radicals, TBARS

## Abstract

In this study we investigated the effects of *Caesalpinia decapetala* (CD) extracts on lipid oxidation in ground beef patties. Plant extracts and butylated hydroxytoluene (BHT) were individually added to patties at both 0.1% and 0.5% (*w*/*w*) concentrations. We assessed the antioxidant efficacy of CD by the ferric reducing antioxidant power (FRAP) assay and evaluated their potential as natural antioxidants for meat preservation by thiobarbituric acid reactive substance (TBARS) values, hexanal content, fatty acid composition and color parameters. These were tested periodically during 11 days of refrigerated storage. TBARS levels were significantly lower (*p* ≤ 0.05) in the samples containing plant extracts or BHT than in the non-treated control. In addition, the beef patties formulated with the selected plant extracts showed significantly (*p* ≤ 0.05) better color stability than those without antioxidants. These results indicate that edible plant extracts are promising sources of natural antioxidants and can potentially be used as functional preservatives in meat products.

## 1. Introduction

Lipid oxidation, one of the major causes of quality deterioration, is also important because it can negatively affect sensory attributes such as color, texture, odor, and flavor as well as the nutritional quality of the product. Meat mincing, cooking and other processing prior to refrigerated storage disrupt muscle cell membranes facilitating the interaction of unsaturated lipids with pro-oxidant substances such as non-hemeiron, accelerating lipid oxidation leading to rapid quality deterioration and development of rancidity. Initially lipid oxidation in meat products results in a cardboard flavor and progresses with the development of painty, rancid and oxidized flavors [1].

Antioxidants are substances that at low concentrations retard the oxidation of easily oxidizable biomolecules, such as lipids and proteins in meat products, thus improving the shelf life of products by protecting them from deterioration caused by oxidation [2].

Synthetic antioxidants such as butylated hydroxyanisole (BHA), butylated hydroxytoluene (BHT), tert-butylhydroquinone (TBHQ), and propyl gallate (PG) have been used as antioxidants in meat and poultry products, but synthetic antioxidants have fallen under scrutiny due to potential toxicological effects [2].

Natural extracts have been developed in response the recent demand for natural products and consumers’ willingness to pay significant premiums for natural foods. Many plants have been recognized as possessing antioxidant activity, including barks of cinnamon (*Cinnamomum iners*), buds of clove (*Syzygium aromaticum* Linn), rhizomes of ginger (*Zingiber officinale* Rosc.), leaves of green tea (*Camellia sinensis*) and leaves of thyme (*Thymus vulgaris* Linn.) [3].

*C. decapetala* (Roth) Alston is a climbing shrub that belongs to the genus *Caesalpinia* of the Fabaceae family. *C. decapetala* (Roth) Alston is widely distributed around the world, but mainly distributed in the southern regions of the Yangtze River in China. The plant is locally known as “Yan wang ci” in Guizhou Province, China. The roots of *C. decapetala* (Roth) Alston are used in folk medicine to treat bronchitis, prevent colds, and as an antimalarial agent. Previous chemical investigations on *C. decapetala* (Roth) Alston revealed that the main chemical components were terpenoids and flavonoids [4]. Recently, the chemical constituents of *C. decapetala* (Roth) Alston have been systematically investigated and the antitumor activities of the compounds have been tested to validate the medicinal use of *C. decapetala* (Roth) Alston. *C. decapetala* has been shown to contain antioxidants. The leaves of *C. decapetala* contain cassane diterpenoid, caesaldecan, spathulenol, 4,5-epoxy-8(14)-caryophyllene, squalene, lupeol, resveratrol, quercetin, astragalin and stigmasterol [5].

Our objective in this study was therefore to evaluate the effectiveness of *C. decapetala* extract in preventing or reducing lipid oxidation as well as color changes in ground beef patties during storage at a chilled temperature (4 °C).

## 2. Results and Discussion

### 2.1. Antioxidant Capacity Assays (AOC)

**Figure 1:**
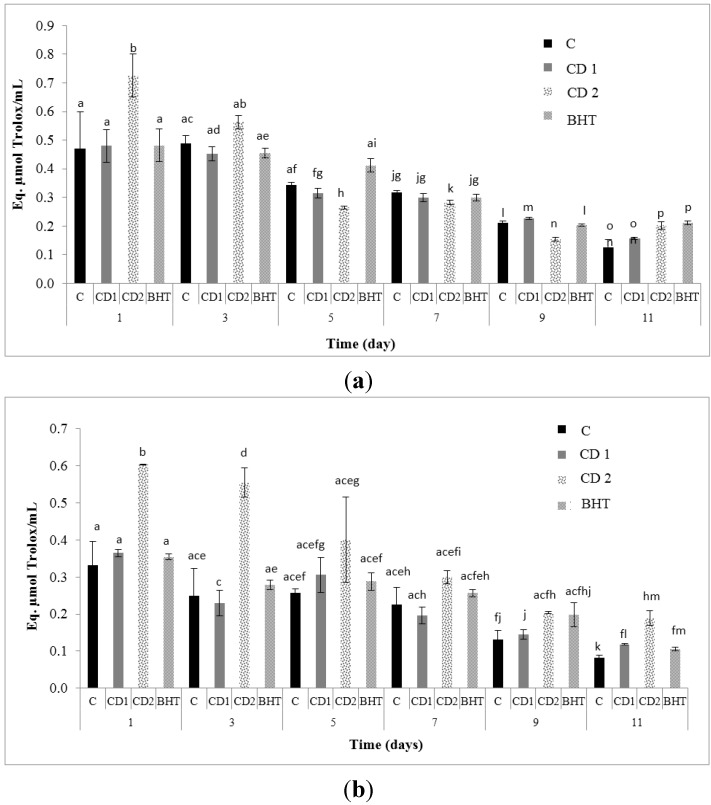
(**a**) AOC measured by FRAP water; (**b**) and FRAP lipid; assay for each treatment: Control (C), CD1 (0.1%), CD2 (0.5%) and BHT after 11 days of storage. The values represent mean ± standard error; Treatment means that do not share a common letter are different (*p* < 0.05).

AOC determined by the ferric reducing antioxidant power (FRAP) assay at 24 h and after 11 days are presented in Figure 1. In order to obtain an accurate value for the total antioxidant activity (Table 1), both the hydrophylic and lipophilic antioxidant activity analyses were done on the same samples.

**Table 1:** The antioxidant activities of *C.* *decapetala* (0.1% and 0.5%) and BHT in beef patties after 11 days of storage.

Sample	Antioxidant Activities (µmol Trolox/mL Sample)		
	Hydrophylic	Lipophilic	Total
Control	0.13 (0.03) ^a^	0.08 (0.01) ^a^	0.21 (0.19) ^a^
CD1	0.16 (0.003) ^b^	0.12 (0.002) ^b^	0.28 (0.07) ^b^
CD2	0.20 (0.01) ^c^	0.19 (0.02) ^c^	0.39 (0.03) ^c^
BHT	0.21 (0.01) ^c^	0.11(0.004) ^b^	0.32 (0.01) ^d^

Results of sample concentrations (µmol Trolox/mL sample) are expressed as mean (SD). Different letters (a–d) in the same column denote significant differences among samples (*p* ˂ 0.05).

The hydrophylic and lipophilic antioxidant activity values were higher in the sample containing *C. decapetala* leaf extract (0.5%). The hydrophylic antioxidant activity (0.20 ± 0.003 mol Trolox equivalent/mL sample) had a higher value than the lipophilic FRAP value with no significant difference (*p* ˂ 0.05) from the sample of BHT (0.21 ± 0.01 mol Trolox equivalent/mL sample). The sample with the lowest antioxidant activity as expected was the control.

The FRAP value is a measure of the capacity of the antioxidant to reduce Ferric (III) ions to Ferrous (II) ions [6]. In our study the final hydrophylic and lipophilic values of antioxidant activity of the sample containing *C. decapetala* (0.5%) were higher than those reported by Topuz *et al.* [7]. They studied the effect of addition of sauces containing olive oil and pomegranate juice into marinated anchovy to retain the initial quality, by preventing undesired chemical and oxidative alterations during storage at 4 °C. The total antioxidant activity value of CD2 (0.39 ± 0.03) was similar to those (0.31 ± 0.05) reported by Bubonja-Sonje *et al.* [8].

The antioxidant activity of the hydrophylic and lipophilic extracts can be attributed to different antioxidants. The hydrophylic extract contains antioxidants such as phenolic derivatives of benzoic acid (gallic acid) and cinnamic acid or flavonoids [9]. In the lipophilic extract the major contributors to the antioxidant activity are hydrophobic compounds such as carotenoids, tocopherols, polymeric proanthocyanidins and high molecular weight tannins [10].

The extracts showed a higher ability to reduce Fe^3+^. The assay showed higher AOC values in the assay carried out with the lipophilic extract compared to the values for the hydrophylic extract.

### 2.2. Effects on Metmyoglobin Formation

**Figure 2:**
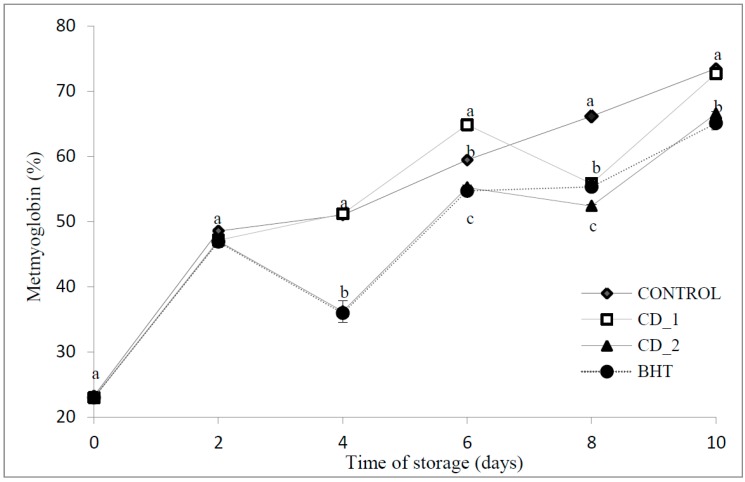
Effects of *C.* *decapetala* extract added at 0.1% and 0.5% (*w*/*w*) and BHT on metmyoglobin changes in beef patties during 11 days of refrigerated storage at 4 °C. Results are given as mean ± standard error. Different letters in the same day (a–c) indicate significant differences between samples.

The effect of *C.* *decapetala* and BHT on MetMB percentage in beef patties is presented in Figure 2. The relative MetMb percentage increased with time for the 11 days of refrigerated storage. The samples treated with leaf extract and BHT had a lower (*p* < 0.05) concentration of MetMb compared to the control, thus demonstrating some ability to inhibit formation of MetMb. After 10 days, the control sample exhibited higher MetMb concentration (73.48 ± 0.20). No significant difference was found between the control and sample CD 1 0.1%. Antioxidant effect was best in samples containing leaf herb extract (66.57% ± 0.3% for *C. decapetala* at 0.5%). The sample with BHT had a very similar behavior to the CD2 extract, with no significant difference between these samples at the end of the study.

Although many factors can influence the color stability of meat and meat products, the susceptibility of myoglobin to autoxidation is a predominant factor. The discoloration of meat from red to brown during storage results from the oxidation of OxyMb to MetMb [11].

The radical species produced during muscle phospholipid oxidation may act to promote OxyMb autoxidation. Conversely, superoxide anion released from oxidized OxyMb can dismutate to hydrogen peroxide and hydroxyl radical, which are potent lipid pro-oxidants [11]. The free radical scavenging effects of phenolic compounds occurring in *C. decapetala* leaf extract are the most likely reason for the retardation of MetMb formation.

In a previous study, Sánchez *et al.* [12] reported that beef patties treated with rosemary did not exceed 40% of metmyoglobin after day 8 of storage. Significant correlations (95%) were observed between metmyoglobin formation and values from the thiobarbituric acid reactive substance (TBARS) assay. This confirms that both parameters reflect the oxidation rate for the samples during the study period, showing the control as the most oxidized sample. The addition of 0.5% *C. decapetala* was effective in inhibiting myoglobin oxidation and maintained the redness of the beef patties due to its ability to maintain oxymyoglobin stability, and to reduce the formation of metmyoglobin.

### 2.3. Volatile Compounds

**Figure 3:**
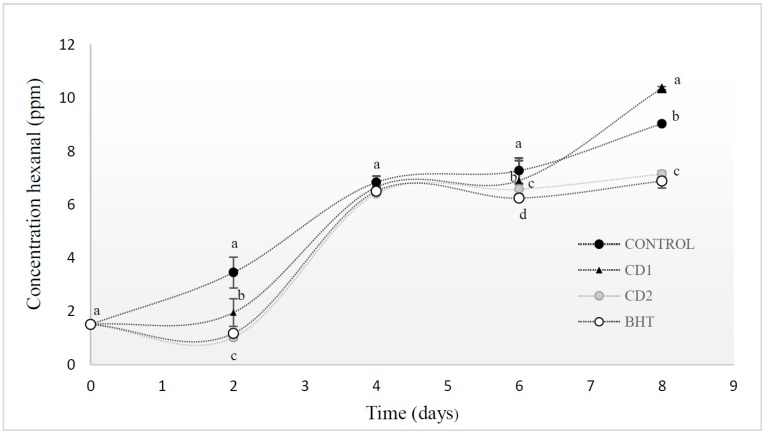
Hexanal in beef patties with *C. decapetala* extract added at 0.1% and 0.5% (*w*/*w*) concentration and BHT packaged in a modified atmosphere and stored at 5 °C. Results are given as mean ± standard error. Different letters in the same day (a–d) indicate significant differences between samples.

The hexanal content increased together with the TBARS values, thereby suggesting lipid oxidation development (Figure 3). The hexanal content of meat stored at 4 °C increased rapidly over the first four days of storage. The trend observed for hexanal values was as follows (*p* ˂ 0.05): control ˃ CD1 ˃ CD2 = BHT. The antioxidant herb extract added to beef patties reduced the amounts of volatile compounds formed. After eight days, the control and CD1 sample showed the highest hexanal concentration throughout the storage period. CD2 (0.5%) extract and BHT samples, which also had the lowest TBARS values, formed the least volatiles with 7.16 ± 0.1 and 6.89 ± 0.1 ppm hexanal, respectively.

Flavor and aroma compounds found in meat include a broad array of compounds, including hydrocarbons, aldehydes, ketones, alcohols, furans, thiophenes, pyrroles, pyrazines, oxazoles, thiazoles, and sulfurous compounds. Also, flavor and aroma are attributes most easily detected and assessed by consumers as either acceptable or not [13].

Aldehydes are the most prominent volatiles produced during lipid oxidation and have been used to successfully follow lipid oxidation in meat or meat products where they are reported to contribute to the overall off-flavor of oxidized meat. Hexanal is reported to be the most sensitive indicator for lipid oxidation [14]. Hexanal and heptanal are degradation products from the oxidation of long chain polyunsaturated fatty acids *n* − 6, mainly linoleic acid [15]. Long chain polyunsaturated fatty acids are known to be less stable towards oxidation than monounsaturated fatty acids, and the high hexanal values observed in this study can be attributed to degradation of linoleic acid.

Similar observations have been also made by Juntachote *et al.* [16] in cooked ground pork sausages with various added antioxidants. Sampaio *et al.* [17] indicated that natural antioxidants including honey, oregano and sage exhibited greater antioxidant efficacy than that shown by BHT, when assessed by hexanal formation.

### 2.4. Effect on Lipid Oxidation and the Color of Beef Patties

#### 2.4.1. Thiobarbituric Acid Reactive Substance (TBARS) Value

**Figure 4:**
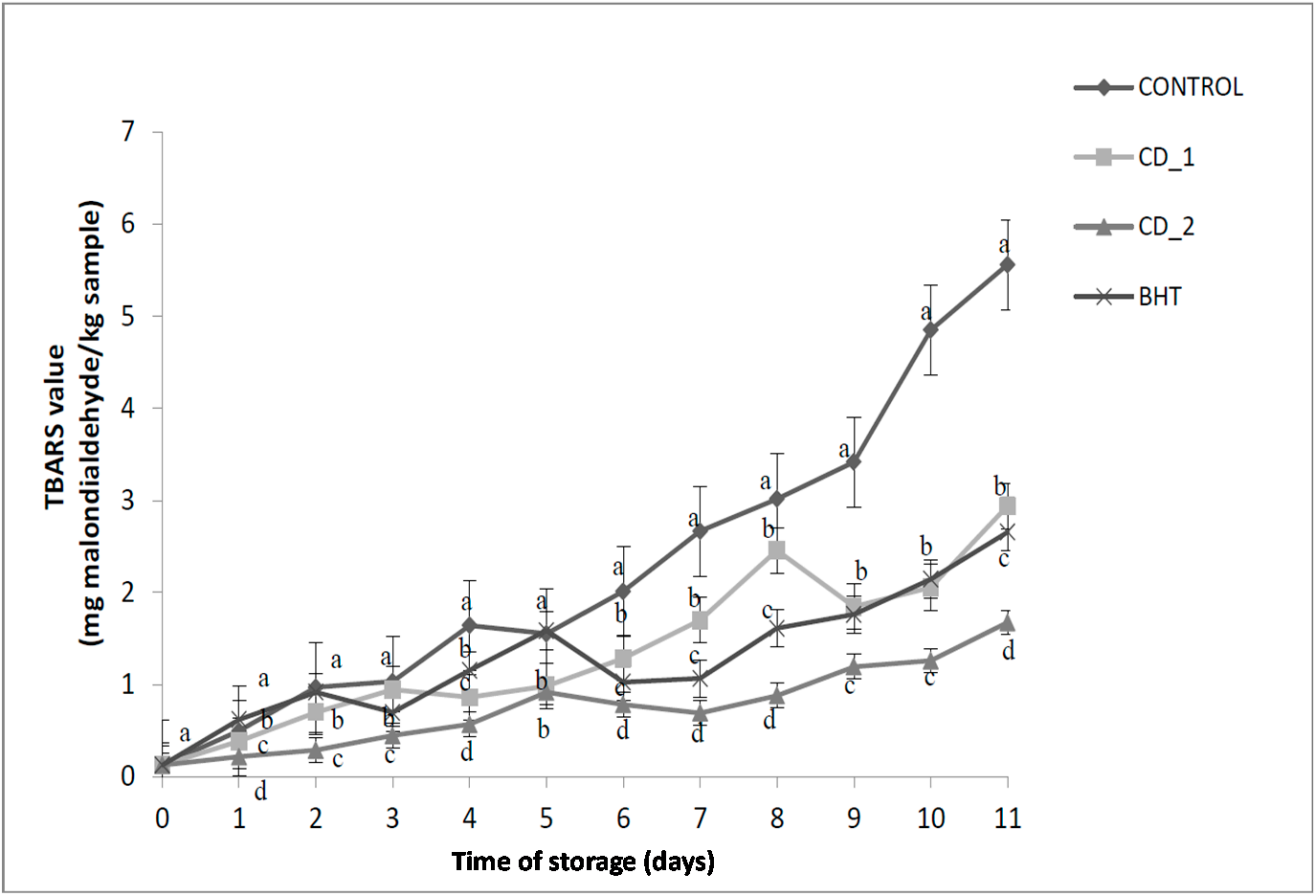
Effects of two concentrations of *Caesalpinia decapetala* added at 0.1% and 0.5% (*w*/*w*) and BHT added at 0.01% (*w*/*w*) on TBARS value (mg of malondialdehyde equivalent/kg of sample) of raw beef patties during 11 days of refrigerated storage at 4 °C. Results are given as mean ± standard error. Different letters in the same day (a–d) indicate significant differences between samples.

The antioxidant effects of *C. decapetala* leaf extracts and the synthetic antioxidant BHT in ground beef patties (0.1% and 0.5%, *w*/*w*) are shown in Figure 4. The extracts showed effective antioxidant activity against lipid oxidation, although the TBARS content of the patties treated with edible plant extract (0.5%) was lower than that of the patties treated with BHT. As expected, the TBARS values of the control sample increased most by 5.6 mg malondialdehyde/kg sample after 11 days, whereas the TBARS values of patties containing 0.1% and 0.5% *C. decapetala* extract increased by 2.9 and 1.7 mg malondialdehyde/kg sample, respectively, after 11 days—significantly less than the control (*p* < 0.05).

The ethanolic extract of *C. decapetala* was moderately antioxidant at both 0.1% and 0.5% in beef patties, with significantly lower (*p* *<* 0.05) TBARS values than the control, and the concentration 0.5% was more effective as an antioxidant than BHT treatment. We concluded that a 0.5% *C. decapetala* leaf extract is more capable than BHT of maintaining lipid stability and efficiently delaying lipid oxidation in refrigerated beef patties.

Our results are consistent with various other studies, all of which reported that natural antioxidants from culinary herbs and edible plants were effective at controlling lipid oxidation and extending the shelf life of meat products. Fasseas *et al.* [18] reported that both oregano essential oil (3%) and sage essential oil (3%) significantly reduced oxidation. Mitsumoto *et al.* [19] reported that adding tea catechins (200 or 400 mg/kg) to minced meat inhibited lipid oxidation in both raw and cooked beef. Similar results to ours were reported by McCarthy *et al.* [20], where an addition of rosemary extracts (0.2%) to beef patties stored in refrigeration had antioxidant activities similar to BHA/BHT (0.01%/0.1%).

Formanek *et al.* [21] noted that rosemary extracts worked synergistically with vitamin E to inhibit the formation of malondialdehyde (TBARS). Han and Rhee [22] showed that 0.25% (*w*/*w*) extracts of rosemary, sappanwood, and red or white peony almost completely inhibited lipid oxidation in raw beef patties.

In general, the effectiveness of these natural antioxidants is proportional to the number of –OH groups present on the aromatic rings. If their solubility is compatible with a particular meat system, the fact that they are natural and have antioxidant activity that is as good as or better than the synthetics makes them particularly attractive for meat products.

#### 2.4.2. Changes in pH of Raw Beef Patties

**Figure 5:**
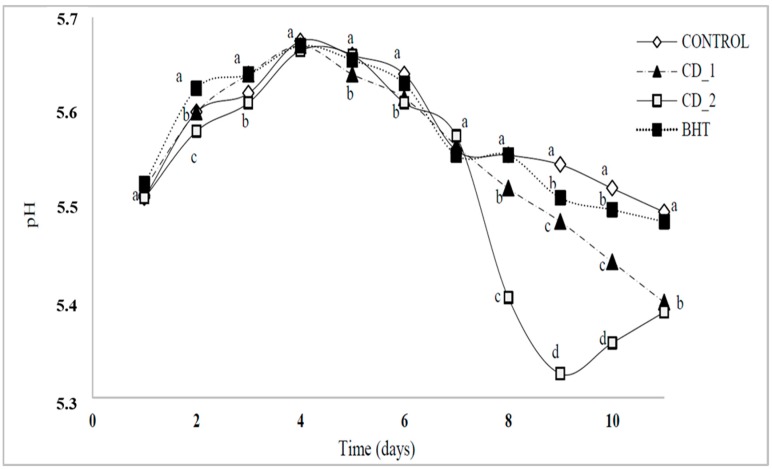
Effects of *Caesalpinia Decapetala* added at 0.1% and 0.5% (*w*/*w*) and BHT added at 0.01% (*w*/*w*) on the changes in pH values of raw beef patties. Data is presented as mean ± standard deviation. Different letters at the same time (a–d) indicate significant differences between samples.

Figure 5 shows the effects of *C.* *decapetala* added at two concentrations (0.1% and 0.5%) *w*/*w* and BHT (0.1%) on the pH values in raw beef patties during cold storage for 11 days. The control sample had the highest pH value (5.50), and the pH values of the other treatments decreased with storage time. Samples treated with *C.* *decapetala *(0.5%) had the lower pH value after storage (5.39). The changes in pH value during storage might be due to acidity produced by bacterial action on the muscle glucose and accumulation of the microbial metabolites due to bacterial spoilage in pork meat patties [22].

#### 2.4.3. Color Changes

The CIE color values in raw ground beef samples with/without spice extracts are shown in Table 2. The color of meat and meat products after slaughter and manufacturing is altered by increased metmyoglobin.

The formation of metmyoglobin is associated with the oxidation of oxymyoglobin (light pink color) during storage. L* values showed a small difference for all samples throughout the storage period. Different authors refer to these slight changes in the values of L* in meat through the storage time [23,24]. The a* value (redness) is the most important color parameter in evaluating meat oxidation, as a decrease in redness makes the meat product unacceptable to consumers. In all samples, the redness (a* value) decreased as storage time progressed. At the end of the study period (Day 11), the intensity of each color parameter was lower than the value measured at Day 0 as a result of the oxidation process, leading in this way to a change in color. It is clear that the protective effects of the test extracts against the color loss (a* value decrease) in stored beef patties were not as pronounced as their effects against lipid oxidation. At the end of storage, the a* values of the CD1 and CD2 samples (−1.02 ± 0.33) were significantly lower (*p* < 0.05) than those of others samples. BHT displayed the highest value of a* at the end of the experiment. Therefore, the natural plant extracts affected meat color, specifically redness, and are therefore potentially useful in prolonging the shelf life of the meat product. Several authors have reported an a* value decrease in different meat and meat products stored under a modified atmosphere [24,25]. The samples had an initial yellowness (b*) value of 3.33 ± 0.82. Significant differences (*p* < 0.05) were observed in b* values in all samples throughout storage.

**Table 2:** Color changes of beef patties containing extracts at 4 °C.

Trait	Days	Control		CD1		CD2		BHT	
Lightness (L*)	0	37.53	(3.19) ^a^						
	1	37.84	(3.24) ^a^	38.86	(1.75) ^a^	37.42	(2.47) ^a^	42.42	(5.14) ^b^
	2	36.42	(2.22) ^a^	39.73	(2.18) ^b^	39.35	(1.77) ^b^	38.71	(2.90) ^b^
	3	39.37	(5.18) ^a^	38.74	(3.55) ^a^	40.17	(3.08) ^a^	40.93	(3.41) ^a^
	4	38.47	(5.18) ^a^	40.92	(2.60) ^b,a^	34.53	(2.35) ^c^	38.52	(2.99) ^b^
	5	42.43	(2.66) ^a^	41.34	(3.05) ^a^	36.78	(2.06) ^b^	37.29	(4.37) ^b^
	6	44.33	(3.36) ^a^	41.74	(3.22) ^b^	40.05	(1.74) ^b^	44.23	(2.59) ^a^
	7	44.38	(4.94) ^a,d^	46.36	(2.85) ^b,d^	40.47	(1.76) ^c^	45.94	(1.88) ^d^
	8	42.85	(2.50) ^a^	44.83	(3.30) ^b,a^	40.59	(2.56) ^c^	35.58	(4.99) ^d^
	9	45.71	(3.32) ^a^	48.14	(3.92) ^b,a^	42.17	(2.65) ^c^	44.63	(2.12) ^a^
	10	44.31	(3.35) ^a^	48.68	(2.14) ^b,a^	46.76	(3.76) ^b,a^	43.07	(3.03) ^a,c^
	11	43.80	(3.66) ^a^	50.90	(1.88) ^b^	43.01	(4.15) ^a^	44.46	(7.98) ^a,b^
Redness (a*)	0	3.33	(0.58) ^a^						
	1	5.08	(1.73) ^a^	5.92	(1.88) ^b^	4.33	(1.82) ^c^	6.42	(2.08) ^d^
	2	6.20	(0.63) ^a^	4.63	(1.18) ^b^	2.35	(1.05) ^c^	6.30	(1.34) ^a^
	3	4.41	(1.67) ^a^	6.52	(1.63) ^b^	4.56	(1.03) ^a^	4.20	(1.33) ^a^
	4	5.04	(2.14) ^a^	4.48	(1.73) ^b,a^	2.57	(0.88) ^c^	3.81	(1.23) ^d^
	5	4.15	(0.43) ^a^	2.43	(0.96) ^b^	1.86	(0.59) ^c,b^	0.87	(0.59) ^d^
	6	2.68	(0.87) ^a^	1.75	(0.81) ^b^	0.83	(0.59) ^c^	2.11	(0.29) ^d^
	7	1.24	(0.28) ^a^	0.44	(0.24) ^b^	0.82	(0.45) ^b,a^	0.72	(0.36) ^b,a^
	8	1.48	(0.35) ^a^	0.53	(0.20) ^b^	0.38	(0.29) ^c,b^	0.66	(0.24) ^d,b^
	9	1.27	(0.38) ^a^	0.95	(0.49) ^a,b^	0.80	(0.40) ^a,b^	0.63	(0.27) ^b^
	10	0.58	(0.44)^a^	0.21	(0.19) ^b^	−0.58	(0.50) ^b^	0.50	(0.40) ^a,b^
	11	−0.47	(0.31) ^a^	−0.78	(0.34) ^b^	−0.70	(0.27) ^a,b^	0.41	(0.23) ^a,b^
Yellowness (b*)	0	3.33	(0.82) ^a^						
	1	6.57	(1.73) ^a^	6.89	(2.45) ^a^	6.44	(2.17) ^a^	8.20	(1.85) ^b^
	2	8.21	(2.08) ^a^	6.46	(2.56) ^b^	8.39	(1.07) ^a^	7.00	(2.01) ^c^
	3	8.15	(2.86) ^a^	10.50	(1.75) ^b^	8.71	(1.47) ^a^	5.89	(2.05) ^c^
	4	6.62	(2.36) ^a,b^	7.38	(2.28) ^a,b^	8.48	(2.43) ^a^	5.56	(2.68) ^b^
	5	8.34	(0.97) ^a^	7.38	(2.50) ^b^	7.60	(1.61) ^b,a^	3.32	(2.25) ^c^
	6	9.60	(2.01) ^a^	10.25	(1.99) ^b,a^	6.17	(1.04) ^c^	9.11	(1.83) ^a,b^
	7	7.02	(3.36) ^a^	6.75	(2.09) ^b^	9.49	(2.27) ^c^	9.00	(1.61) ^c^
	8	10.46	(0.98) ^a^	9.89	(1.55) ^a^	9.55	(1.30) ^a^	2.90	(2.00) ^b^
	9	7.76	(2.59) ^a^	8.74	(2.99) ^b^	8.56	(1.42) ^b^	7.39	(1.97) ^a^
	10	7.69	(1.16) ^a^	8.90	(1.87) ^b,a^	11.59	(3.01) ^c^	9.70	(1.50) ^d,b^
	11	4.52	(1.35) ^a^	12.30	(2.42) ^b^	10.57	(3.04) ^c,b^	6.23	(4.06) ^d,a^

Results of color changes are expressed as mean (SD). Means with different letters (a–d) in the same day are significantly different at *p* < 0.05.

## 3. Experimental Section

### 3.1. Plant Material

*C. decapetala* was collected from Perú in the spring of 2012. This was crushed and stored (inside a desiccator) in the dark, at room temperature until use.

### 3.2. Chemicals

Reagents used were: 2-thiobarbituric acid, Trolox, gallic acid, ethanol, phosphate buffered saline (PBS), anhydrous sodium carbonate, were purchased from Sigma-Aldrich Química S.A. (Madrid, Spain) and, methanol, hydrogen chloride, ferrous chloride (FeCl_2_) were acquired from Panreac Química S.L.U. (Barcelona, Spain). Distilled water and Milli-Q (Millipore, Barcelona, Spain) water were extracted daily.

### 3.3. Sample Extraction

The powder (1.5 g) of the dried leaves of *C. decapetala* was stirred using a magnetic stirrer with 25 mL mixture of ethanol/water (5:5) for 24 h at 4 °C. The extraction was done in triplicate. Then the mixture was centrifuged at 2500 rpm and the separated liquid was collected. A portion of the separated liquid was stored at −80 °C until use to determine the antiradical capacity, and the remaining part was concentrated in a rotary evaporator and lyophilized. The lyophilized extract was stored in a desiccator until use.

### 3.4. Preparation of Beef Patties

Fresh beef samples were purchased from a local fresh food market and minced three times. The meat was mixed with salt (1.5%). Four formulations were prepared: negative control (without antioxidant), CD1 (*C. decapetala* 0.1% *w*/*w*), CD2 (*C. decapetala* 0.5%) and BHT (0.01%). The mixing process for each formulation was replicated. The ingredients were mixed manually in a steel bowl for about 1 min to obtain a homogeneous mixture, and then burgers were formed manually. The beef patties were randomly selected and packed in plastic trays which were filled with a gas mixture of 70% O_2_ + 20% CO_2_ + 10% N_2_ before sealing and they were kept refrigerated at 4 °C for 11 days.

### 3.5. Antioxidant Capacity Assay (AOC)

AOC was determined by the ferric reducing antioxidant power (FRAP) assay. The sample preparation for AOC studies involved extraction of *C. decapetala* with distilled water or a lipophilic extraction solvent system (acetone/ethanol/distilled water; 5:4:1, *v*/*v*/*v*, according to Linden [26].). These solvent systems were used to extract the hydrophylic and lipophilic antioxidants. The determination of the antioxidant capacity of antioxidants extracted was performed by the FRAP assay (ferric reducing antioxidant power). Based on this, the methods are called FRAPwater, or FRAPlipid assays.

Prior to extraction, deep frozen muscle samples were minced by a disintegrator. Muscle homogenate (5 g) was weighed into a glass tube and mixed with 5 mL of extraction solvent for 30 s using an Ultra-Turrax. Two different extractions were performed. The first was made with distilled water to dissolve the hydrophylic antioxidants and the second was carried with an acetone/ethanol/water mixture (5:4:1, *v*/*v*/*v*) to dissolve the lipophilic antioxidants in the muscle. Then the samples were centrifuged at 4 °C for 30 min. Supernatants were filtered using a folded filter and stored in the dark and on ice until immediate analysis. Once the supernatant was obtained, the FRAP assay was performed. Measurements were carried out in triplicate.

#### FRAP Assay

The FRAP assay was carried out according to the procedure described by Benzie and Strain [6] with minor modification The determination of reducing capacity was performed with microplates, mixing the FRAP reagent incubated at 37 °C with the samples (in an appropriate dilution to cause the absorbance to fall in the range 0.1–1.0). The FRAP reagent was prepared from sodium acetate buffer (300 mM, pH 3.6), 10 mM TPTZ solution (40 mM HCl as solvent) and 20 mM iron (III) chloride solution in a volume ratio 10:1:1, respectively. The samples were measured in triplicate. The absorbance of the reaction mix was then detected at 593 nm. The results were expressed as milimoles of Trolox equivalents/g of dry plant.

### 3.6. Determination of Metmyoglobin

Patty samples (5 g) were homogenized together with 25 mL of 0.04 M phosphate buffer (pH 6.8) for 10 s using an Ultra-Turrax mixer. The homogenate was allowed to stand for 1 h at 4 °C and centrifuged at 4500 *g* for 20 min at 4 °C using a high-speed cooling centrifuge. The absorbance of the filtered supernatant was read at 572, 565, 545, and 525 nm. The percentage of metmyoglobin was determined using the formula of Krzywicki [27]:

MetMb (%) = [2.514 (*A*_572_/*A*_525_) + 0.777 (*A*_565_/*A*_525_) + 0.8 (*A*_545_/*A*_525_) + 1.098] × 100

### 3.7. Headspace Volatile Analysis

Hexanal was measured using a TRACE GC gas chromatograph equipped with a mass spectrometer DSQII (Thermo Fisher Scientific) with automatic injector TRIPLUS with a *Head Space* module*.* One gram of homogenized patties was placed in a 10 mL glass vial capped with an aluminum cap with PTFE/silicone septum. The sample was shaken and heated at 60 °C for 30 min in an autosampler heating block before measurement. Vapor phase (1 mL) was injected using a special gas syringe maintained at 65 °C. Hexanal concentrations were determined from peak areas using a standard curve prepared from authentic hexanal.

### 3.8. Determination of Secondary Oxidation by TBARS

The TBARS method was used to measure the lipid oxidation over the storage period as described by Grau *et al.* [28].The TBARS reagent was prepared from 15% trichloroacetic acid, 0.375% thiobarbituric acid and 2.1% hydrochloric acid. Briefly, 1 g of sample was weighed and protected with 1 mL aqueous EDTA, and was mixed with 5 mL of thiobarbituric acid reagent using an Ultra-Turrax (IKA, Staufen, Germany) at 32,000 rpm speed for 1 min. It was then filtered with a Whatman filter (0.45 μm) to obtain the part soluble in the solvent. All procedures were carried out in the dark and all samples were kept on ice. Immediately, the filtered samples were immersed in a water bath preheated to 95 °C ± 1 °C for 10 min. Samples were cooled at room temperature for 10 min and the absorbance was measured at λ = 531 nm. TBARS value was calculated from the calibration curve of malondialdehyde (MDA). The results are expressed as mg malondialdehyde (MDA)/kg of meat.

### 3.9. pH Measurement

The pH of 5 g samples was determined with a pH meter (Mettler-Toledo GLP 21, Schwerzenbach, Switzerland).

### 3.10. Color Measurements

Color measurements were performed at four points on the surface of the patties using a Minolta Chromameter CR-300. Color measurements were observed at 11 days of chilled storage. The L*, a* and b* values (CIE L*, a*, b* color system) were assessed as a measure of lightness, redness and yellowness, respectively. The instrument was calibrated using a black-and-white glass tile, provided with the instrument. The meat samples were kept inside the plastic dishes in triplicate, and then the instrument was directly placed on the surface of the meat at three different points for each plastic dish. For every point result was a mean of nine measurements. The mean and standard error for each parameter were calculated.

### 3.11. Statistical Analysis

The data were reported as means ± standard deviation (SD). Statistical analyses were conducted using the Minitab software program. The significance of differences was determined by the one-way analysis of variance (ANOVA) with Duncan’s pairwise comparison (*p* < 0.05).

## 4. Conclusions

Our experiments with raw beef patties indicated that the *C. decapetala* extract may be promising as a source of natural antioxidants for meat products. The analysis of TBARS, fatty acid degradation, antioxidant activity and concentration of volatile compounds provides a complete assessment of the consequences of lipid oxidation in burger patties. The addition of this extract to the beef patties at 0.5% was the most effective antioxidant. This concentration inhibited formation of TBARS and volatile compounds more effectively than the synthetic antioxidant BHT over the course of 11 days. Using this herb extract as an ingredient in burger patties may be an efficient strategy to enhance the nutritional value and safety of these meat products.

## References

[1] Lagerstedt A., Lundstrom K., Lindahl G. (2011). Influence of vacuum or highoxygen modified atmosphere packaging on quality of beef *M. longissimus* dorsi steaks after different ageing times. Meat Sci..

[2] Jayathilakan K., Sharma G.K., Radhakrishna K., Bawa A.S. (2007). Antioxidant potential of synthetic and natural antioxidants and its effect on warmed-over-flavour in different species of meat. Food Chem..

[3] Hinneburg I., Dorman H.J.D., Hiltunen R. (2006). Antioxidant activities of extracts from selected culinary herbs and spices. Food Chem..

[4] Zhang Q., Liu X.T., Liang J.Y., Min Z.D. (2008). Chemical constituents from the stems of *Caesalpinia decapetala*. Chin. J. Nat. Med..

[5] Kiem P.V., Minh C.V., Huong H.T., Lee J.L., Kim Y.H. (2005). Caesaldecan, a cassane diterpenoid from the leaves of *Caesalpinia decapetala*. Chem. Pharm. Bull..

[6] Benzie I.F.F., Strain J.J. (1996). The ferric reducing ability of plasma (FRAP) as a measure of “antioxidant power”: The FRAP assay. Anal. Biochem..

[7] Topuz O., Yerlikaya P., Ucak I., Gumus B., Aydan H. (2014). Effects of olive oil and olive oil-pomegranate juice sauces on chemical oxidative and sensorial quality of marinated anchovy. Food Chem..

[8] Bubonja-Sonje M., Giacometti J., Abram M. (2011). Antioxidant and antilisterial activity of olive oil, cocoa and rosemary extract polyphenols. Food Chem..

[9] Servili M., Selvaggini R., Esposto S., Taticchi A., Montedoro G., Morozzi G. (2004). Heaalth and sensory properties of virgin olive oil hydrophilic phenols: Agronomic and technological aspects of production that affect their ocurrence in the oil. J. Chromatogr. A.

[10] Bianco A., Uccella N. (2000). Biophenolic components of olives. Food Res. Int..

[11] Lindahl G. (2011). Colour stability of steaks from large beef cuts aged under vacuum or high oxygen modified atmosphere. Meat Sci..

[12] Sánchez-Escalante A., Djenane D., Torrescano G., Beltrán J.A., Roncalés P. (2001). The effects of ascorbic acid, taurine, carnosine and rosemary powder on colour and lipid stability of beef patties packaged in modified atmosphere. Meat Sci..

[13] MacLeod G., Shahidi F. (1994). The flavour of beef. Flavor of Meat and Meat Products.

[14] Im S., Hayakawa F., Kurata T. (2004). Identification and sensory evaluation of volatile compounds in oxidized porcine liver. J. Agric. Food Chem..

[15] Meynier A., Genot C., Gandemer G. (1998). Volatile compounds of oxidized pork phospholipids. J. Am. Oil Chem. Soc..

[16] Juntachote T., Berghofer E., Siebenhandl S., Bauer F. (2007). The effect of dried galangal powder and its ethanolic extracts on oxidative stability in cooked ground pork. LWT Food Sci. Technol..

[17] Sampaio G.R., Salsanha T., Soares R.A.M., Torres E.A.F.S. (2012). Effect of natural antioxidant combinations on lipid oxidation in cooked chicken meat during refrigerated storage. Meat Sci..

[18] Fasseas M.K., Mountzouris K.C., Tarantilis P.A., Polissiou M., Zervas G. (2008). Antioxidant activity in meat treated with oregano and sage essential oils. Food Chem..

[19] Mitsumoto M., O’Grady M.N., Kerry J.P., Buckley D.J. (2005). Addition of tea catechins and vitamin C on sensory evaluation, colour and lipid stability during chilled storage in cooked or raw beef and chicken patties. Meat Sci..

[20] McCarthy T.L., Kerry J.P., Kerry J.F., Lynch P.B., Buckley D.J. (2001). Assessment of the antioxidant potential of natural food and plant extracts in fresh and previously frozen pork patties. Meat Sci..

[21] Formanek Z., Kerry J.P., Higgins F.M., Buckley D.J., Morrison P.A., Farkas J. (2001). Addition of synthetic antioxidants to α-tocopherol acetate supplemented beef patties: Effects of antioxidants and packaging on lipid oxidation. Meat Sci..

[22] Han J., Rhee K.S. (2005). Antioxidant properties of selected Oriental non-culinary/nutraceutical herb extracts as evaluated in raw and cooked meat. Meat Sci..

[23] Bingol E.B., Ergun O. (2011). Effects of modified atmosphere packaging (MAP) on the microbiological quality and shelf life of ostrich meat. Meat Sci..

[24] Esmer O.K., Irkin R., Degirmencioglu N., Degirmencioglu A. (2011). The effects of modified atmosphere gas composition on microbiological criteria, color and oxidation values of minced beef meat. Meat Sci..

[25] Cachaldora A., García G., Lorenzo J.M., García-Fontán M.C. (2013). Effect of modified atmosphere and vacuum packaging on some quality characteristics and the shelf-life of “morcilla”, a typical cooked blood sausage. Meat Sci..

[26] Linden S. (2003). α-Tocoperol, β-Carotin und l-Ascorbinsäure in der Rindermast—Einfluss auf Qualitätsparameter. Ph.D. Thesis.

[27] Krzywicki K. (1982). The determination of heam pigments in meat. Meat Sci..

[28] Grau A., Guardiola F., Boatella J., Barroeta A., Codony R. (2000). Measurement of 2-thiobarbituric acid values in dark chicken meat through derivative spectrophotometry: Influence of various parameters. J. Agric. Food Chem..

